# Mushroom *Bulgaria inquinans* Modulates Host Immunological Response and Gut Microbiota in Mice

**DOI:** 10.3389/fnut.2020.00144

**Published:** 2020-10-08

**Authors:** Hongzhen Sang, Yu Xie, Xing Su, Mengdi Zhang, Yijie Zhang, Kun Liu, Junpeng Wang

**Affiliations:** ^1^Institute of Infection and Immunity and Translational Medical Center, Huaihe Hospital of Henan University, Kaifeng, China; ^2^School of Basic Medical Science, Henan University, Kaifeng, China; ^3^School of Physical Education, Henan University, Kaifeng, China; ^4^Department of Respiration, The First Affiliated Hospital of Henan University, Kaifeng, China; ^5^College of Biology Science and Engineering, Hebei University of Economics and Business, Shijiazhuang, China

**Keywords:** mushroom, *Bulgaria inquinans*, prebiotic, immune responses, mesenteric lymph node, gut microbiota

## Abstract

We aimed to determine the prebiotic impact of Mushroom *Bulgaria inquinans* (BI) on the host immune response and gut microbiota. Male C57BL/6 mice were fed a diet supplemented with 0, 1, or 2% BI for 4 wks. Compared to mice fed with a control diet (0% BI), mice fed with 1 or 2% BI had an increase of T cell proliferation from the spleen, but such change was not found between 1 and 2% BI treated mice. Also, BI at 2% increased the production of IL-2 of splenocytes stimulated with T-cell mitogens, but BI at 1 and 2% did not affect productions of other splenic-T cell cytokines including IL-4, IL-10, and IFN-γ. Interestingly, BI at 1 or 2% inhibited T cell proliferation of mesenteric lymph node (mLN) but this effect was not found between 1 and 2% BI treated mice. Furthermore, BI inhibited the production of IL-2 in anti-CD3/CD28-stimulated T cells from mLN in a dose-dependent manner. Meanwhile, BI at 2%, not 1% inhibited the production of IL-4, IL-10, and IFN-γ of mLN. Since BI at 2% produced a more significant effect on the immune response, we further used BI at 2% to evaluate the effect of BI on gut microbiota. Of note, BI reduced the diversity of gut microbiota and resulted in an increase of *Faecalibaculum* and *Parabacteroides* abundance and the decrease of *Allobaculum, Candidatus*_*Saccharimonas*, and *Rikenella* abundance at the genus level. Finally, the correlation was observed between specific bacteria genera and the productions of T-cell cytokines from mesenteric lymphocytes: *Rikenella* and *Candidatus_Saccharimonas* correlated positively with IL-2, IL-4, IL-10, and IFN-γ; *Bacteroides* and *Parabacteroides* correlated negatively with IL-2 and IL-4; *Faecalibaculum* correlated negatively with IFN-γ and IL-4 and *Bacteroides* and *Bifidobacterium* correlated negatively with IFN-γ. The specific role of each intestinal microbiota observed is still unclear, but BI might exert a prebiotic effect on gut microbiota by increasing the abundance of potentially beneficial bacteria (*Faecalibaculum*). This is helpful for further demonstrating the healthy-promotion mechanism of *B. inquinans*.

## Introduction

Both innate and adaptive immune functions play crucial roles in preventing and controlling pathogenic infection, neoplasia, and maintaining immune homeostasis in the body. Currently, there are limited approaches available to efficiently modulate the immune response. Nutritional interventions that involve optimizing the intake of essential nutrients and using potential functional foods have become an increasingly popular strategy to regulate the function of immune cells. Edible mushrooms, containing many bioactive components such as polysaccharides, glycoproteins, proteins, lipids, and secondary metabolites, have been explored extensively for their immunomodulating properties including enhanced NK activity (1), promoted dendritic cell maturation and functions (2), augmented vaccine efficacy to protect against infection (3), and anti-obesity (4) in animal models. Besides, the immunomodulating properties including improved γδ- and NK- T cell proliferation and activation, increased IgA production in healthy young adults (5), and anti-inflammatory effects in ulcerative colitis and Crohn's disease (6, 7) are observed in edible mushrooms.

The non-lichenized ascomycete *Bulgaria (B.) inquinans* is an edible wood-inhabiting ascomycete growing on freshly felled oak and widely found in the area of Changbai Mountain (Northeastern of China). The fruit bodies of *B. inquinans* are delicious food after treated by Na_2_CO_3_ solution. However, eating too much can cause lip-swelling or valgus, which can be due to photosensitive dermatitis. Because of this swelling, it is sometimes referred to as the “pig-mouth mushroom.” *B. inquinans* has been used as food and traditional antitumor medicine for many years (8). Also, evidence shows that several compounds isolated from the fruit bodies of *B. inquinans* have been demonstrated to have antibacterial (9), antitumor (10), antipruritics and antierythema effects (11). Importantly, polysaccharides which exist in the fruit bodies of *B. inquinans* have been isolated and purified and then demonstrated to have antioxidant activity *in vitro* (12) and the immunological activities including ConA- and LPS-induced lymphocyte proliferation *in vivo* (13).

Gut microbiota plays a crucial role in regulating the systemic immune function during health and diseases (14). Whereas, gut microbial ecology can be modulated through diet (15, 16). For example, calorie restriction diets (low fat- or carbohydrate diets) could result in weight loss by the increase of the prevalence of Bacteroidetes (17). In addition to the effect of macronutrients on altering the gut microbiota, a deficiency in micronutrients could alter the gut microbial communities in the gut (18, 19). Although dietary fiber is not digestible by the mammalian host, it can be readily digested by the intestinal microbiota (20, 21). Therefore, dietary intervention is a potential tool to regulate gut microbial ecology and have applications in maintaining the intestinal homeostasis, preventing and treating chronic diseases associated with microbial dysbiosis.

In recent years, studies performed utilizing animal models have shown that consuming some medicinal (22, 23) and edible mushrooms have the health promoting-prebiotic effect (24, 25). Especially, the β-glucans found in edible mushrooms such as white button mushroom or *B. inquinans* could enhance immune function via stimulating toll-like receptors on immune cells (13). Edible mushrooms may support healthy immunity and affect inflammatory responses through interaction with the gut microbiota (26), which indicated that some of the benefits of edible mushrooms may profit from their impact on the microbiota. Although oral supplementation with polysaccharide isolated from *B. inquinans* is effective in modulating certain immune functions (27), little is known about the potential immunological and microbiota effects of fruit bodies of *B. inquinans*. The current study aimed to investigate the effects of *B. inquinans* feeding on host immune functions including peripheral and mesenteric immune organs and microbiota as well as a possible mechanism of action in mice.

## Materials and Methods

### Diet and Mice

Fruit bodies of *B. inquinans* (BI) were purchased from Tonghua in Jilin Province, China. After its authentication was confirmed by Professor Xiangmei Zhang, a voucher specimen (NoF121733) was deposited in the College of Biology Science and Engineering of Hebei University of Economics and Business for future reference.

After *B. inquinans* was treated with Na_2_CO_3_, the mushroom powder was prepared and added at 1 or 2% (wt:wt) to an AIN-93G diet (Research Diets) (1% BI and 2% BI, respectively) as previously described (3). These doses are considered translationally relevant because they are achievable through the normal dietary intake in humans. The 1% dose for mice can be converted to a daily consumption of 1.1 g fresh mushrooms/kg body weight for humans by using isocaloric calculation (28) or ~75 g fresh mushrooms/d (1 serving) for a person weighing 65–70 kg. Similarly, the 2% mushroom is equivalent to 2 servings/d.

**Figure 1:**
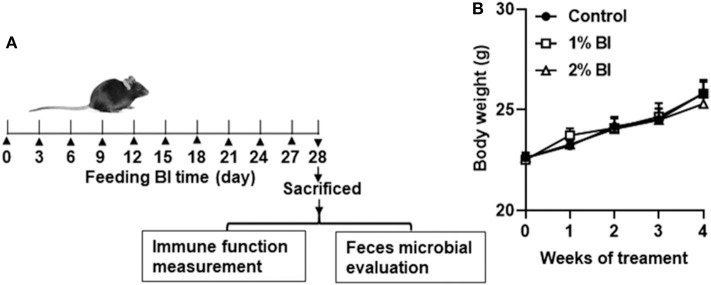
Effect of *B. inquinans* supplementation on body weight. **(A)** Flow diagram of this study. **(B)** Body weight changes of the mice. Values are means ± SEM (*n* = 6 mice/group). Control, control diet; BI, *B. inquinans*.

All experimental procedures were conducted following the Guide for the Care and Use of Laboratory Animals by the National Institute of Health (NIH Publications No. 8,023, revised 1,978) and approved by the Institutional Animal Care and Use Committee at Huaihe Hospital of Henan University (No: HHYY2018008). Specific pathogen-free male C57BL/6 mice (6–8 weeks of age) were obtained from Nanjing Biomedical Research Institution of Nanjing University (Nanjing, China) and individually housed in a controlled environment (temperature 23°C, relative humidity 45%) with a 12:12 h light: dark cycle. Mice were provided with free access to water and the experimental diet *ad libitum*. Mice were divided randomly into three experimental treatment groups (Figure 1A). The control group was fed a control diet and the 1% and 2% group was fed 1% and 2% BI, respectively. Body weight was measured weekly until the end of the intervention.

### Determination of Cytokines and T Cell Proliferation

After feeding for 4 wks, mice (*n* = 6/group) were euthanized with CO_2_ asphyxiation followed by exsanguination. The spleens and mesenteric lymph nodes (mLN) were aseptically collected and single-cell suspensions were prepared as previously described (3), and viable cells were determined and counted using trypan blue exclusion. Splenocytes and mesenteric lymphocytes were, respectively, seeded into 24-well culture plate at 3 × 10^6^/well in the presence of plate-coated anti-CD3 (5 μg/ml) and soluble anti-CD28 (1 μg/ml) mAbs (anti-CD3/CD28) or ConA (1.5 μg/ml) for 48 h to determine cytokine productions of IFN-γ, IL-2, IL-4, and IL-10 by T cells using ELISA kits (all from BD Pharmingen).

Furthermore, after splenocytes and mesenteric lymphocytes were stimulated with anti-CD3/CD28 for 72 h, cells were collected to determine T cell proliferation by measuring the ki-67 expression in total CD3^+^ T cells using a Mindray Bricyte E6 flow cytometry (Shenzhen, China).

### Cell Subpopulations

To determine the percentage of total T cells (CD3), macrophages (F4/80), and NK cells (NK1.1), splenocytes and mesenteric lymphocytes were blocked with anti-CD16/CD32 mAb (Fc block from eBioscience, San Diego, CA) and then multi-stained with fluorescence-conjugated anti-CD3 (T cells), anti-F4/80 (macrophages), and anti-NK1.1 (NK cells) mAbs (all from eBioscience). To determine the T cell proliferation, anti-CD3/anti-CD28-stimulated cells were blocked and then multi-stained with fluorescence-conjugated anti-CD3 and anti-ki-67 (a cellular proliferation marker) mAbs (eBioscience) using the Foxp3/Transcriptional Factor Staining Buffer Set (eBioscience). All antibodies and reagents were from eBioscience. Data acquired on a Mindray BriCyte® E6 flow cytometer (Shenzhen, China) were analyzed using FlowJo 10.0 software (Tree Star).

### Microbiota Analysis

Murine feces were collected on day 28, immediately frozen in liquid nitrogen, and stored at −80°C until DNA extraction. Total genome DNA was extracted using the CTAB/SDS method. The V3-V4 region of 16S rRNA genes was PCR-amplified by using a specific primer (341F-806R) with Barcode (Supplementary Figure 1). All PCR reactions were performed in 30 μL reactions with 15 μL of Phusion® High-Fidelity PCR Master Mix (New England Biolabs), 0.2 μM of forward and reverse primers and 10 ng template DNA. Thermal cycling consisted of initial denaturation at 98°C for 1 min, followed by 30 cycles of denaturation at 98°C for 10 s, annealing at 50°C for 30 s, and elongation at 72°C for 30 s, followed by a final step of 72°C for 5 min, and sequenced on an Ion S5TM XL platform by Wuhan Servicebio Technology Co. Ltd (Wuhan, China).

Sequence analysis of the clean reads for all samples was performed by UPARSE software (UPASE v7.0.1001) at ≥97% sequence similarity cut off following with SILVA with a confidence score threshold of 80% using Mothur software to obtain taxonomic information and its relative abundance. To study the phylogenetic relationship of different Operational Taxonomic Units (OTUs), and the difference of the dominant species in different samples (groups), multiple sequence alignment was conducted using the MUSCLE software (Version 3.8.31) (29).

### Statistics Analysis

Data in the tables or figures were presented as means ± SEM. Statistical analysis was carried out by one-way ANOVA test followed by a Bonferroni *post hoc* test for multiple comparisons or non-paired Student's *t*-test using GraphPad Prism 8.0 software. Alpha diversity (observed-species, Chao, Ace, Shannon, and Simpson) was performed with the QIIME (Version1.7.0) and the data were displayed with the R software (Version 2.15.3). To explore the key gut flora which may be related to the inflammation of the gut, Spearman's correlation analyses were performed among the known gut genera and the production of cytokines secreted by mesenteric lymphocytes, and Spearman's correlation analyses were performed using GraphPad Prism 8.0 software. Significance was set at *P* < 0.05.

## Results

### Body Weight

During the whole experiment, all mice in each diet group remained healthy. Daily food intake of group pair-fed mice was from 2.9 to 3.5 g. No difference in body weight was found among the diet groups at the start or the end of the study (Figure 1B).

### Cell Subpopulations in Spleen and Mesenteric Lymph Node

**Figure 2:**
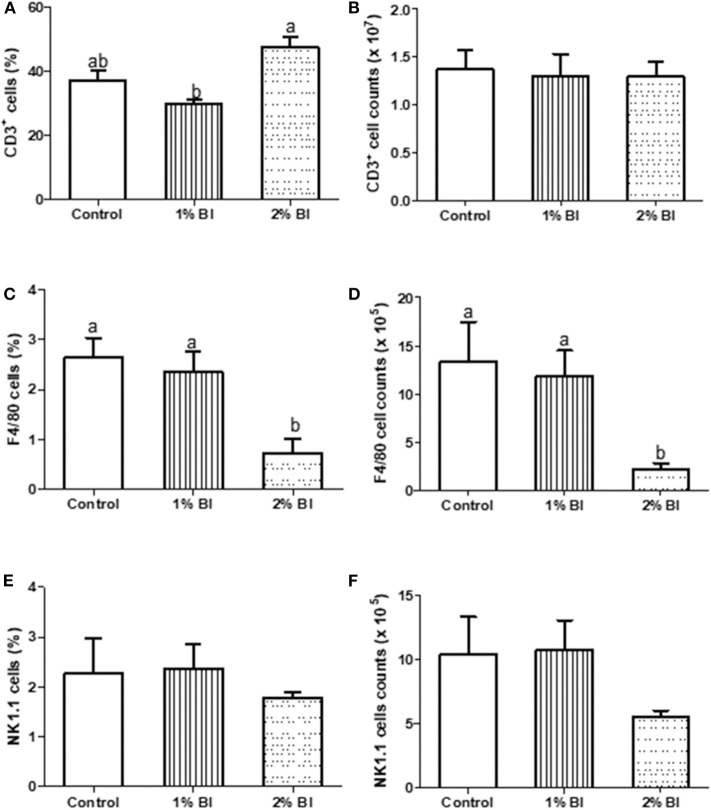
Effect of *B. inquinans* supplementation on splenocyte populations. On day 28, mice were sacrificed with CO_2_ and spleen was collected to determine the immune cell populations including T cells [CD3, **(A,B)**], macrophages [F4/80, **(C,D)**] and NK cells [NK1.1, **(E,F)**] by flow cytometry described as the “Materials and Methods” section. Values are means ± SEM (*n* = 6 mice/group). Means without a common letter differ at *P* < 0.05. Control, control diet; BI, *B. inquinans*.

**Figure 3:**
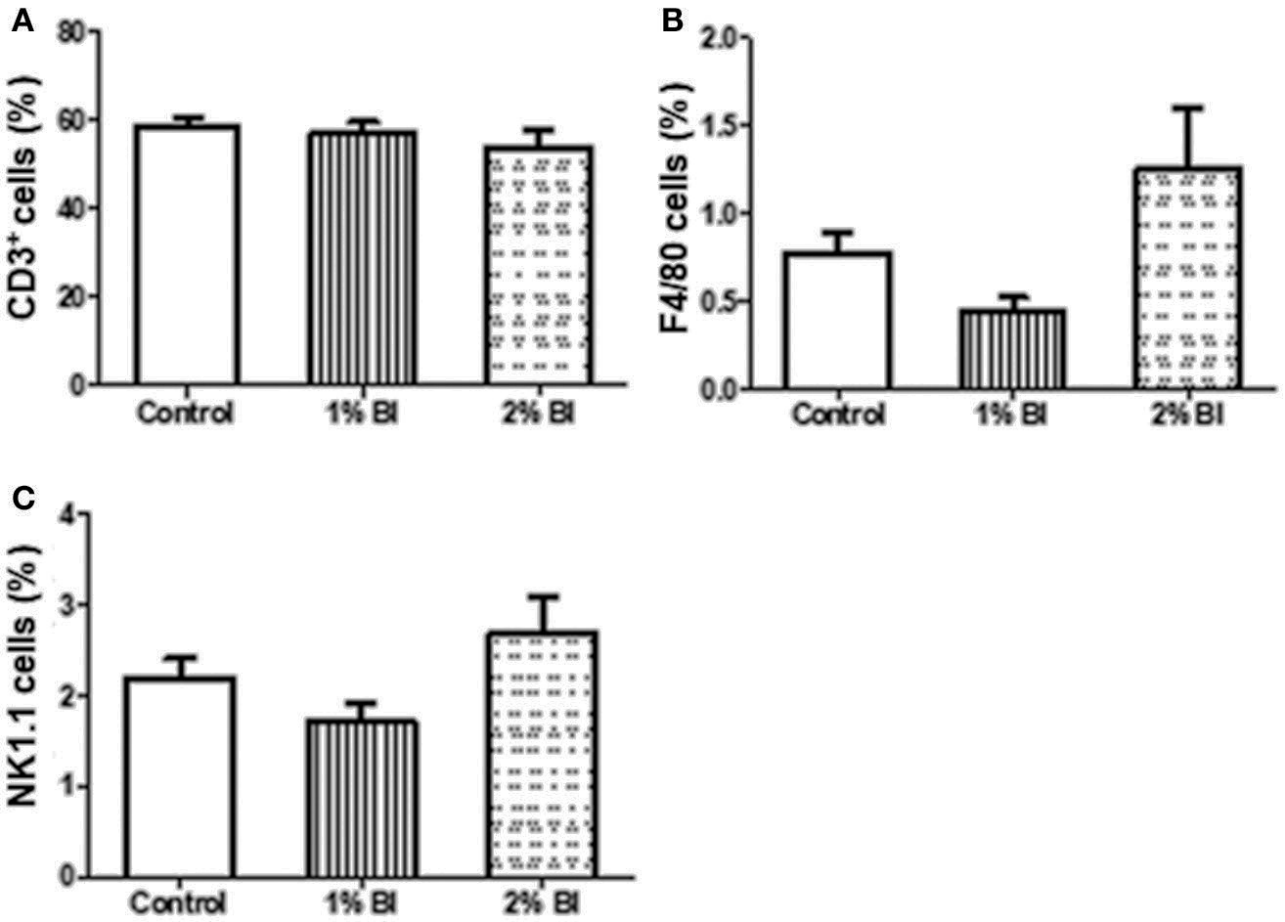
Effect of *B. inquinans* supplementation on mesenteric lymphocyte populations. On day 28, mice were sacrificed with CO_2_ and all mesenteric lymphocyte populations was collected and pooled to determine the immune cell populations including T cells [CD3, **(A)**], macrophages [F4/80, **(B)**] and NK cells [NK1.1, **(C)**] using flow cytometry described as the section Materials and Methods. Values are means ± SEM (*n* = 6 mice/group). Control, control diet; BI, *B. inquinans*.

The percentage and numbers of total T cells (CD3^+^) (Figures 2A,B) and NK cells (NK1.1^+^) (Figures 2C,D) in the spleen did not differ among the 3 diet groups. However, the percentage and number of Mø (F4/80) in mice fed with the 2% BI were lower than those of mice fed the control and 1% BI diet (Figures 2C,D). In the mesenteric lymph node, the percentage of total T cells, NK cells, and Mø did not differ among the 3 diet groups (Figure 3).

### Cytokine Production in Spleen and Mesenteric Lymph Node

In the spleen, mice fed with the 2% BI diet had the highest amounts of IL-2 secreted by T cells stimulated either with anti-CD3/CD28 or ConA than those of mice fed with the control and 1% BI diet (Table 1). IFN-γ (Th1 cytokine), IL-4, and IL-10 (Th2 cytokines) production did not differ among the different diet groups (Table 1).

**Table 1:** Effect of BI supplementation on cytokine production of splenocytes stimulated with T-cell mitogens.

**Cytokines**	**Diet**		
	**Control**	**1% BI**	**2% BI**
**IL-2 (pg/ml)**			
Anti-CD3/CD28	888 ± 235^a^	830 ± 142^a^	1933 ± 220^b^
ConA	24 ± 10^a^	33 ± 20^a^	122 ± 35^b^
**IFN-γ** **(ng/ml)**			
Anti-CD3/CD28	65.4 ± 5.5	65.59 ± 8.3	48.92 ± 6.5
ConA	12.93 ± 2.90	15.55 ± 3.59	17.77 ± 4.24
**IL-4 (pg/ml)**			
Anti-CD3/CD28	306 ± 43	614 ± 260	969 ± 290
ConA	ND	ND	ND
**IL-10 (pg/ml)**			
Anti-CD3/CD28	14.36 ± 1.34	17.20 ± 0.85	16.12 ± 1.10
ConA	326 ± 108	538 ± 94	555 ± 91

*Values are means ± SEM (n = 6 mice/group). Different letters indicate a statistically significance (P < 0.05). ND, Non-detectable; Control, control diet; BI, B. inquinans*.

In the mesenteric lymph node, anti-CD3/CD28-stimulated secretion of IL-2 was significantly lower in the group fed with the 1% BI diet than in the control diet group and was further lessened in the 2% BI diet group (Table 2). In addition, the 2% BI-fed mice had significantly lower IFN-γ, IL-4, and IL-10 production compared to the control group (Table 2). However, no difference for IFN-γ, IL-4, and IL-10 production was found between the control and 1% BI diet group (Table 2).

**Table 2:** Effect of BI supplementation on cytokine production of mesenteric lymphocytes stimulated with T-cell mitogen (Anti-CD3/CD28).

**Cytokines**	**Diet**		
	**Control**	**1% BI**	**2% BI**
IL-2 (ng/ml)	4.36 ± 0.30^a^	2.14 ± 0.31^b^	0.39 ± 0.05c
IFN-γ (ng/ml)	54.71 ± 6.90^a^	38.66 ± 6.53^a^	11.54 ± 5.84^b^
IL-4 (pg/ml)	177 ± 9^a^	143 ± 26^a^	48 ± 10^b^
IL-10 (ng/ml)	7.06 ± 2.46^a^	7.82 ± 2.29^a^	0.71 ± 0.16^b^

*Values are means ± SEM (n = 6 mice/group). Different letters indicate a statistically significance (P < 0.05). Control, control diet; BI, B. inquinans*.

### T Cell Proliferation in Spleen and Mesenteric Lymph Node

**Figure 4:**
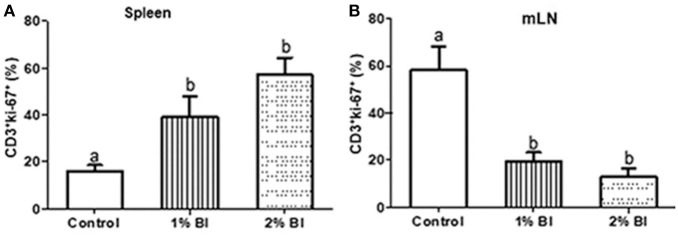
Effect of *B. inquinans* supplementation on T cell proliferation from splenocytes and mesenteric lymphocytes. On day 28, splenocytes and mesenteric lymphocytes were stimulated with plated-coated anti-CD3 (5 μg/ml) and soluble anti-CD28 (1 μg/ml) Abs to evaluate T cell proliferation pregated on CD3^+^ using flow cytometry. Values are means ± SEM (*n* = 6 mice/group). Means without a common letter differ at *P* < 0.05. Control, control diet; BI, *B. inquinans*.

In the spleen, T cell proliferation was significantly increased in the 1 or 2% BI diet group compared to the control group, but no difference in such changes was found between the 1 and 2% BI groups (Figure 4A). Contrary to splenic T cell proliferation, mice fed with the 1 or 2% BI diet showed an inhibitory effect on T cell proliferation of mesenteric lymphocytes when compared with the control mice, but no difference in such changes was found between the 1 and 2% BI treated groups (Figure 4B).

### Gut Microbiota

A total number of 90 7,200 raw sequences were obtained with an average of 75,600 tags per mouse. After sequence processing, 847,008 clean reads of 16S rRNA (accounting for 93.40% of the raw reads) with an average length of 404 bp were obtained to be used for further analysis. The sequences were clustered into OTUs with 97% similarity cut off, and a total of 724 OTUs were obtained. Then the OTUs for bacteria were categorized into 23 phyla, 31 classes, 62 orders, 97 families, 136 genera, and 68 species by making a comparison to a SILVA database.

**Figure 5:**
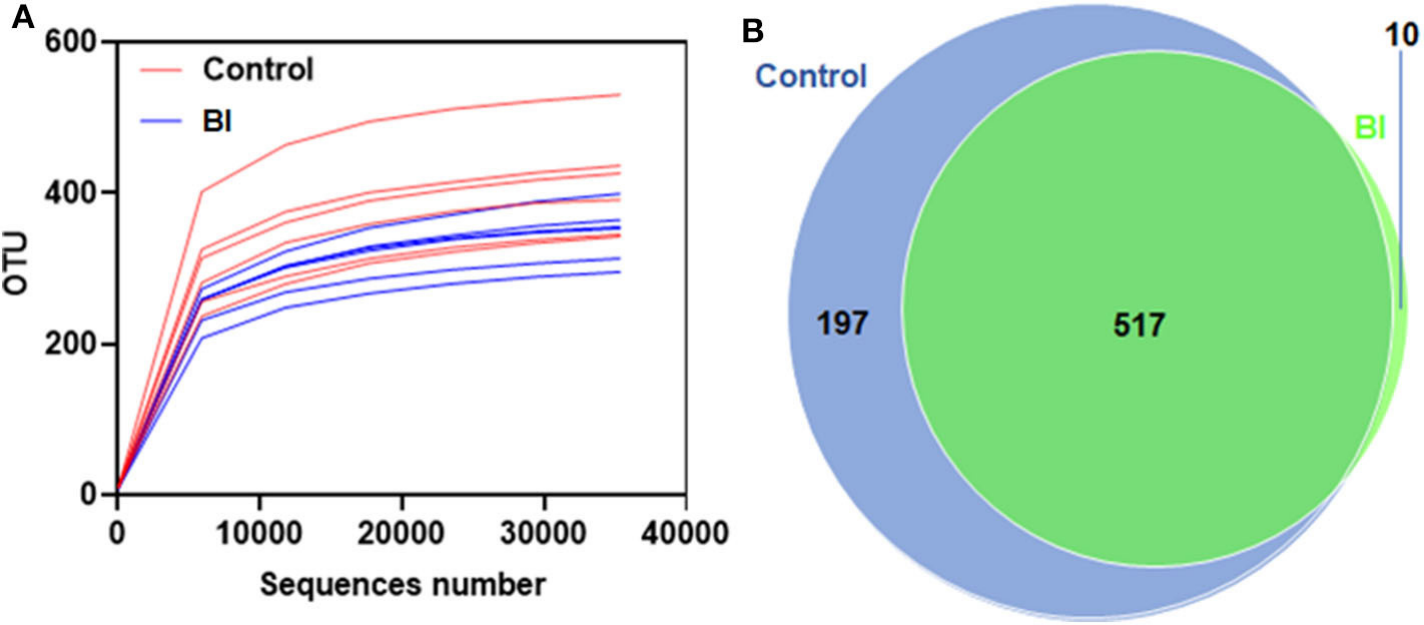
Rarefaction curves for the control group and 2% BI administration group. On day 28, the feces were collected to evaluate the gut microbiota using unique OTUs. **(A)** Rarefaction curves showing unique OTUs at the 97% threshold. **(B)** Venn Graph showing common and unique OTUs in the control group and 2% BI administration group. Control, control diet; BI, *B. inquinans*.

The rarefaction curve is used to determine whether the current sequencing depth of each sample can be enough to reflect the microbial diversity. As shown in Figures 5A,B, with the number of sequences increased, the number of identified OTUs reached saturation of the rarefaction curves, which demonstrated that the samples used in this study were commendably able to reflect the diversity of the bacterial richness and diversity that could be used for the following difference analysis. Meanwhile, comparisons of OTUs showed an overall similar richness of OTUs between control and BI-treated mice at the 3% similarity level (Figure 5B). Furthermore, the Venn diagram shows the common and unique OTUs between two group samples (Figure 5B). The percentages of OTUs unique to the Control and BI groups were 27.2, and 1.39%, respectively, and 71.4% OTUs were shared by two groups, which suggested the similarity in the bacterial structure between the two groups.

**Figure 6:**
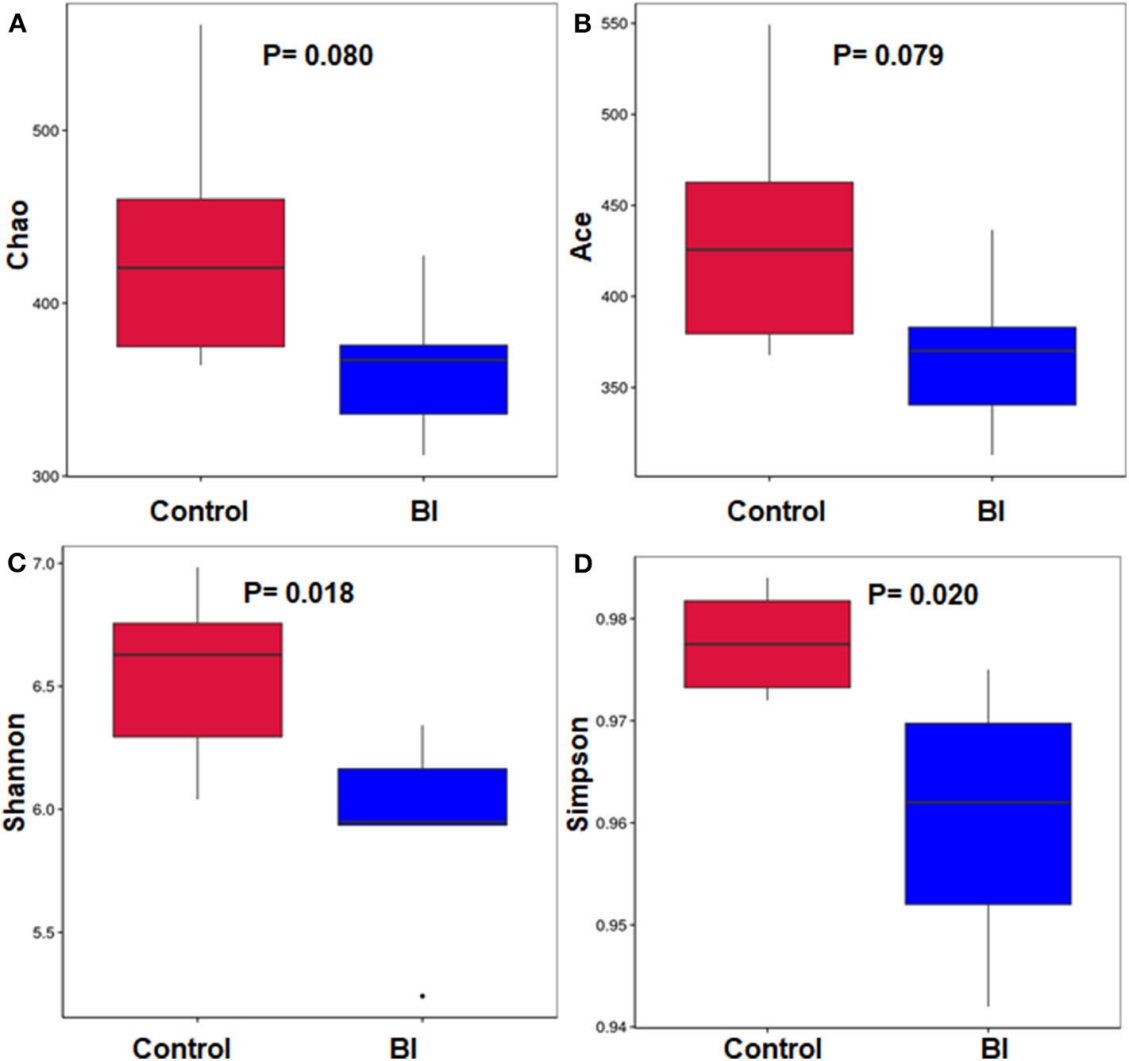
The richness and diversity of the bacterial community in the control group and 2% BI administration group. **(A)** Chao index; **(B)** Ace index; **(C)** Shannon index; **(D)** Simpson index. Control, control diet; BI, *B. inquinans*.

As an alternative approach of bacteria richness and diversity assessment, we determined the Chao, Abundance-based Coverage Estimator (Ace), Shannon, and Simpson diversity. In consistent with the rarefaction curve data from the sequenced OTUs, feeding BI had a reduced trend in either Chao or Ace index between these two groups (Figures 6A,B). Further, the Shannon and Simpson diversity indices of the fecal microbiome were evaluated in these two groups. As shown in Figures 6C,D, dietary BI reduced both diversity indices (*p* < 0.05). These results suggested that feeding BI could reduce the richness and diversity of the microbiome.

**Figure 7:**
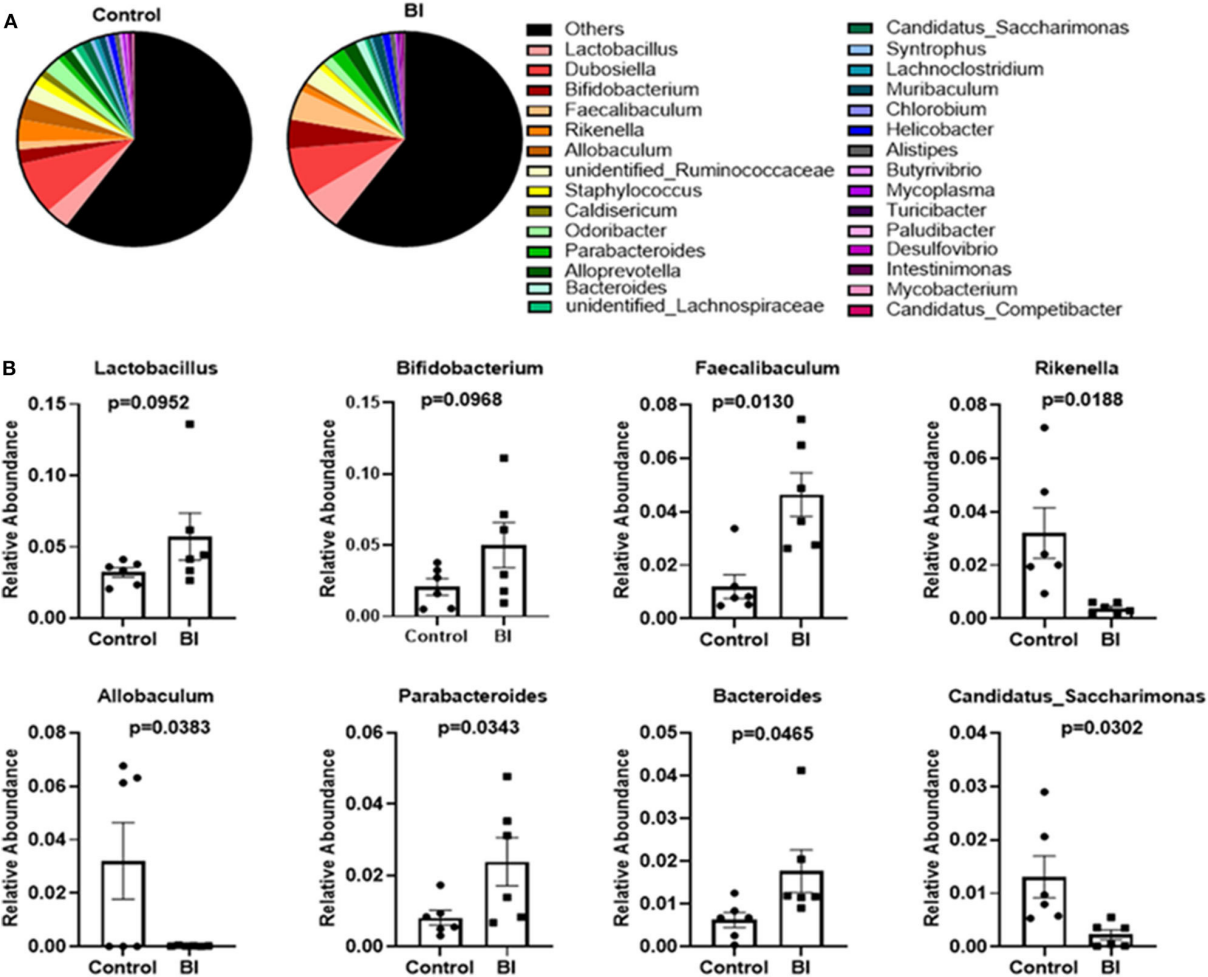
The changes in the relative abundance at the genus level of the gut microbiota of mice in the control group and BI treatment group at day 28. **(A)** The relative abundance of the top 30 genera between two groups. **(B)** BI affected the specific bacterial genera. Values are means ± SEM (*n* = 6 mice/group). Control, control diet; BI, *B. inquinans*.

There was no significant change at the phylum, class, order, or family levels after 4 wks feeding between control and BI groups (Data not shown). At the genus level, we performed the phylogenetic relationship of genus horizontal species between the control and BI groups through multiple sequence alignment. Figure 7 displays the relative abundance of the top 30 genera among the two different groups. At the genus, BI supplementation increased the relative abundance of *Faecalibaculum* and *Parabacteroides* and decreased the relative abundance of *Allobaculum, Candidatus*_*Saccharimonas*, and *Rikenella*. Also, BI supplementation showed a tendency toward increased SCFA-producing bacteria (*Bifidobacterium*) and lactic acid-producing bacteria (*Lactobacillus*). These distinct responses of individual genera to BI suggests that BI might profoundly impact the gut microbial community.

### Correlation Between Specific Gut Genera and Mesenteric Cytokine Level

**Figure 8:**
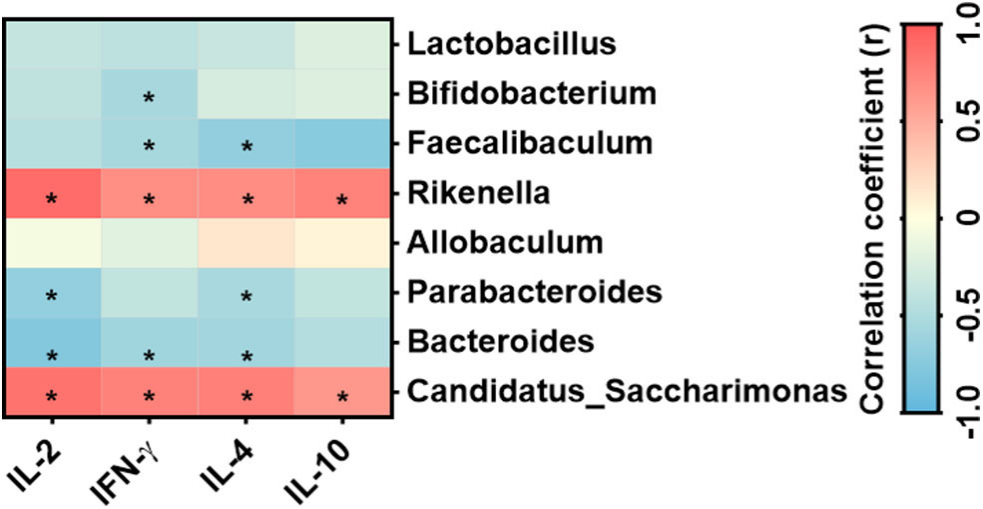
Correlation between the specific bacterial genera and cytokines secreted by mesenteric lymphocytes *n* = 6 mice/group. *Statistical significant based on a *P* < 0.05 (Spearman's correlation coefficient). The heat map was generated using Prism 8.0 software.

Figure 8 revealed that the cytokines IL-2, IL-4, IL-10, and IFN-γ correlated positively with *Rikenella* and *Candidatus_Saccharimonas*; while *Bacteroides* and *Parabacteroides* correlated negatively to a significant degree with IL-2 and IL-4. Moreover, IFN-γ secreted by mesenteric lymphocytes significantly correlated negatively with *Faecalibaculum, Bacteroides*, and *Bifidobacterium*, and *Faecalibaculum* correlated negatively with IL-4 produced by mesenteric lymphocytes.

## Discussion

Studies show that the dietary intake of a variety of mushrooms, their extracts and isolated compounds are effective in modulating some aspects of immune function (30), which is crucially important to eliminate self-antigens and invading pathogens. Furthermore, consumption of edible mushrooms has the capability of modulating gut microbial populations (31), which in turn may exert pro- and anti-inflammatory responses by regulating the function of immune cells during immune responses (14, 32, 33). Since some components isolated and purified from *B. inquinans* can alter the body's immune response (11, 13), it has been implied that whole *B. inquinans* treatment could influence the body's immune system and gut microbial ecology. To our knowledge, for the first time, this study shows that feeding mice a 4-wk dietary supplementation of mushroom *B. inquinans* affected the body's immune responses, including the peripheral and mucosal immune system by enhancing mitogens-stimulated T cell proliferation and IL-2 production of splenocytes while suppressing mitogen-stimulated T cell proliferation and T-cell secretion of IL-2, IL-4, IL-10, and IFN-γ in mesenteric lymphocytes and the perspective of gut commensal bacteria.

In the human body's immune system, there are several main types of immune cells, such as macrophages, NK cells, and T cells. Mushrooms and their extracted polysaccharides have been shown to possess immunological activities that can protect the body from microbial and parasite attacks via modulating the function of immune cells. e.g., white button mushrooms (WBM) could enhance NK activity, not affect NK cell numbers in C57BL/6 mice (1) and dietary WBM can enhance the efficacy of vaccination against microbial infection (3). Polysaccharides isolated from *B. inquinans* enhanced splenic lymphocyte proliferation in malaria-bearing mice and normal mice (13, 27), which suggest that *B. inquinans* could impact the body's immune functions possibly via its' bioactive components such as polysaccharides. Indeed, *B. inquinans* treatment enhanced the proliferation of splenic T cells when compared with the control diet. In contrast, T cell proliferation of MLN was significantly inhibited by *B. inquinans* treatment. However, which bioactive components such as polysaccharides isolated from *B. inquinans* could affect intestinal immune response needs to be further evaluated.

T cell-mediated immune response is pivotal in modulating both the type and extent of the immune responses. T helper (Th1) cells mainly produce IL-2 and IFN-γ and are crucial to defense against intracellular pathogens infection. Th2 cells mainly secrete IL-4 and IL-10 and are mainly involved in humoral responses to extracellular infection. Polysaccharides from fungi have been demonstrated to modulate the immunological activities such as the induction of Th cell reaction in a different manner. However, it lacks evidence for the effect of *B. inquinans* or its extracted polysaccharides on cytokine production of T cells. Polysaccharides isolated from *B. inquinans* could promote splenic lymphocyte proliferation (13, 27), indicating that *B. inquinans* treatment might have the ability to change cytokine production of immune cells. As expected, *B. inquinans* administration increased the production of IL-2 of mitogens-activated T cells from the spleen while decreasing the production of cytokines including IL-2, IL-4, IL-10, and INF-γ from mLN. IL-2 is a crucial cytokine to promote T cell survival, growth, and expansion (34). These results indicate that *B. inquinans* may promote or suppress T cell proliferation possibly via the enhancement or impairment of IL-2 from the spleen or mLN, respectively. Furthermore, since impairing T cell responses in the mLN, *B. inquinans* may be undesirable in certain microbial infection. This is further supported by our feeding experiment *in vivo* in which *B. inquinans* treatment decreased the survival and increased the burden of bacterial in the liver and spleen in *Salmonella*-infected mice (unpublished results). However, it may be beneficial for certain autoimmune and inflammatory disorders where overactive T-cell mediated response plays a role. Thus, future studies using appropriate animal models are needed to further evaluate the clinical significance of the observed immunomodulatory effects of *B. inquinans*.

It is well-known that gut microbiota plays a central role in the immune homeostasis or the maintenance of a stable immune system (35). A breakdown of intestinal immune homeostasis is a major cause of chronic inflammatory bowel diseases (36). In addition, the gut microbial dysbiosis is implicated in the etiopathogenesis or manifestation of other diseases such as multiple sclerosis and Alzheimer's disease (37).

Therefore, how to maintain the symbiotic relationship that the immune system shares with the microbiota may be a powerful strategy to improve human health. Prebiotics could suppress endogenous pathogens found with the intestinal tract via inducing a stronger immune system against exogenous pathogens (38, 39). The prebiotics are good ingredients (such as mushroom) that are selectively utilized by host microbes which provide various health benefits. Oligosaccharides and fibers are the major sources of prebiotics that can regulate gut microbiota composition with increased numbers of *bifidobacteria* and improve gut barrier function (40, 41). Studies have found that the important sources of prebiotics, which are non-digestible polysaccharides derived from mushrooms and other foods, can have health-promoting effects via enhancing the growth of gut probiotic bacteria (25, 31, 42). *Lentinula edodes*-derived polysaccharide L2 has shown great effects on the gut microbiota and immune-stimulating activity (43) and *Lentinula edodes*-derived β-1 → 3,1 → 6-glucan has the anti-inflammatory activities (44). Although *B. inquinans*-derived (1 → 6)—β-D-glucan has been proved to have the immunological activities such as mitogens (LPS and ConA)-induced lymphocyte proliferation, whether *B. inquinans* treatment could influence the gut microbiota is still unknown. To our knowledge, we first demonstrated that *B. inquinans* reduced the diversity and evenness of gut microbiota in the feces. Consistent with our study, mushroom *Lentinula edodes*-derived polysaccharide could reduce the diversity and evenness of gut microbiota along the intestine (43), indicating that mushroom polysaccharide might regulate gut microbial diversity. Currently, as little is known about the structure of microbiome interaction networks, May's study suggests that species diversity can be problematic for community stability (45). A mathematical analysis from Foster and colleagues predicts that high species diversity leads to unstable microbiome communities (46). Thus, low species diversity might be beneficial to make gut microbial ecology stable. These data suggest that dietary *B. inquinans* could contribute to gut microbiome stability.

*B. inquinans* did not affect the microbial composition at the phylum, class, order, or family levels, but it significantly altered the microbial composition at the genus levels including increasing the abundance of *Faecalibaculum* and *Parabacteroides* and decreasing the abundance of *Allobaculum, Candidatus*_*Saccharimonas*, and *Rikenella*. Importantly, these bacteria were positively or negatively correlated with cytokines secreted by mesenteric lymphocytes. For example, *Faecalibaculum* has been shown to have anti-tumor properties (47) and *Candidatus_Saccharimonas* is involved in maintaining normal intestinal function (48). *Rikenella* is positively correlated with inflammatory factors (49) and correlated with chronic systemic inflammatory disorders (50), suggesting that the *Rikenella* contributes to promoting inflammation. Thus, current data indicate that *B. inquinans* could influence the host immune response possibly via modulating the abundance of these bacteria.

Currently, the specific role of each intestinal microbiota observed in this study is unknown. But our results reported the overall alteration in the structure of intestinal microbiota, which might be due to the direct effect of fungal polysaccharide on enterocytes that can secret cytokines and modulate the immune responses to the gut microbiota (51), which finally shaped the gut microbiota ecology (52). Inflammatory bowel disease is characterized by gut microbiota dysbiosis (53). However, *B. inquinans* showed great effects on gut microbiota and immunological activities. Therefore, further studies are urgently needed to explore the possibility of the preventive and/or therapeutic application of *B. inquinans* to dysbiosis-related diseases.

In the present study, we only explored the effects of *B. inquinans* on the splenocyte and mesenteric lymphocyte functions and the gut microbiota in the feces. Thus, one limitation of this study is the lack of determining the effects of *B. inquinans* treatment on the gut microbiota in the intestines (small intestine, cecum, and colon contents) and the immune response in other gut-associated lymphoid tissues (Peyer's patch and small intestine lamina propria). Cignarella et al. (49) demonstrated that mice fed with the normal diet for 0 and 4 wks did not affect the diversity and richness of gut microbiota community and gut microbiota composition; whereas different diets could significantly change gut microbiota community, suggesting that the normal diet might not affect gut microbiota ecology in a controlled environment in mice. However, to strictly present our data, another limitation of the study is the lack of evaluating whether there is a difference in the immune response and gut microbiota at baseline.

Furthermore, since mushroom *B. inquinans* can be considered toxic in large quantities because of photosensitive dermatitis, it has not been used globally as an edible mushroom (only used as an edible mushroom in China), indicating that it cannot be referred directly to food and special attention should be paid to the preparation of meals with *B. inquinans*.

## Conclusions

In conclusion, feeding *B. inquinans* promoted mitogen-stimulated T cell proliferation and T-cell secreted IL-2 from peripheral immune tissue (Spleen) while inhibited mitogen-stimulated T cell response including T cell proliferation and T-cell related cytokine production from mucosal immune organs. Further gut microbiota analysis demonstrates that *B. inquinans* treatment decreased the diversity and evenness of gut microbiota in the feces. Other significantly changed populations in response to *B. inquinans* treatment include *Faecalibaculum, Parabacteroides, Allobaculum, Candidatus*_*Saccharimonas*, and *Rikenella*. In particular, the abundance of *Candidatus*_*Saccharimonas and Rikenella* are exclusively and positively correlated with all cytokine levels produced by mesenteric lymphocytes in *B. inquinans*-treated mice.

## Data Availability Statement

The datasets generated for this study can be found in NCBI Bioproject, accession no. PRJNA634429.

## Ethics Statement

The animal study was reviewed and approved by Institutional Animal Care and Use Committee at Huaihe Hospital of Henan University.

## Author Contributions

JW, KL, and YZ: conceptualization. HS, YX, XS, and MZ: investigation. JW and HS: writing—original draft preparation. JW, KL, YZ, HS, and YX: writing—review and editing. All authors have read and agreed to the published version of the manuscript.

## Conflict of Interest

The authors declare that the research was conducted in the absence of any commercial or financial relationships that could be construed as a potential conflict of interest.

## References

[1] Wu D Pae M Ren Z Guo Z Smith D Meydani SN. Dietary supplementation with white button mushroom enhances natural killer cell activity in C57BL/6 mice. J Nutr. (2007) 137:1472–7. 10.1093/jn/137.6.1472 17513409

[2] Ren Z Guo Z Meydani SN Wu D. White button mushroom enhances maturation of bone marrow-derived dendritic cells and their antigen presenting function in mice. J Nutr. (2008) 138:544–50. 10.1093/jn/138.3.544 18287364

[3] Wang J Niu X Du X Smith D Meydani SN Wu D. Dietary supplementation with white button mushrooms augments the protective immune response to salmonella vaccine in mice. J Nutr. (2014) 144:98–105. 10.3945/jn.113.185165 24259557

[4] Shimizu T Mori K Ouchi K Kushida M Tsuduki T. Effects of dietary intake of japanese mushrooms on visceral fat accumulation and gut microbiota in mice. Nutrients. (2018) 10:610. 10.3390/nu10050610 29757949 PMC5986490

[5] Dai X Stanilka JM Rowe CA Esteves EA Nieves C Jr Spaiser SJ. Consuming *Lentinula edodes* (*Shiitake*) mushrooms daily improves human immunity: a randomized dietary intervention in healthy young adults. J Am Coll Nutr. (2015) 34:478–87. 10.1080/07315724.2014.950391 25866155

[6] Førland DT Johnson E Sætre L Lyberg T Lygren I Hetland G. Effect of an extract based on the medicinal mushroom *Agaricus blazei* murill on expression of cytokines and calprotectin in patients with ulcerative colitis and Crohn's disease. Scand J Immunol. (2011) 73:66–75. 10.1111/j.1365-3083.2010.02477.x 21129005

[7] Therkelsen SP Hetland G Lyberg T Lygren I Johnson E. Cytokine levels after consumption of a medicinal *Agaricus blazei* murill-based mushroom extract, andosan™, in patients with crohn's disease and ulcerative colitis in a randomized single-blinded placebo-controlled study. Scand J Immunol. (2016) 84:323–31. 10.1111/sji.12476 27588816

[8] Huang NL Colored Illustrations Od Macrofungi (Mushrooms) of China. Beijing: China Agricultural press (1998).

[9] Stadler M Anke H Dekermendijan K Reiss R Sterner O Witt MR New azaphilones from fruit bodies and mycelial cultures of the ascomycete *Bulgaria inquinans*. Fr Nat Prod Lett. (1996) 7:7–14. 10.1080/10575639508043180

[10] Yang XJ Zhang HW Sun H Zhang ZW Kang B Zhang SC The antitumor activity of *Bulgaria inquinans* (fries). Special Wild Econ Anim Plant Res. (1993) 2:9–12.

[11] Jiang S Tsumuro T Takubo M Fujii Y Kamei C. Antipruritic and antierythema effects of ascomycete *Bulgaria inquinans* extract in ICR Mice. Biol Pharm Bull. (2005) 28:2197–200. 10.1248/bpb.28.2197 16327148

[12] Bi H Gao T Li Z Ji L Yang W Jeff Iteku B. Structural elucidation and antioxidant activity of a water-soluble polysaccharide from the fruit bodies of *Bulgaria inquinans* (fries). Food Chem. (2013) 138:1470–5. 10.1016/j.foodchem.2012.11.039 23411269

[13] Bi H Gao T Liu D Tai G Wei M Zhou Y. Structures of (1–>6)-beta-D-glucans from *Bulgaria inquinans* (fries) and their immunological activities. Carbohydr Polym. (2013) 93:547–52. 10.1016/j.carbpol.2012.12.036 23499095

[14] Lee YK Mazmanian SK. Has the microbiota played a critical role in the evolution of the adaptive immune system? Science. (2010) 330:1768–73. 10.1126/science.1195568 21205662 PMC3159383

[15] Ooi JH Waddell A Lin YD Albert I Rust LT Holden V. Dominant effects of the diet on the microbiome and the local and systemic immune response in mice. PLoS ONE. (2014) 9:e86366. 10.1371/journal.pone.0086366 24489720 PMC3906035

[16] Dalby MJ Ross AW Walker AW Morgan PJ. Dietary uncoupling of gut microbiota and energy harvesting from obesity and glucose tolerance in mice. Cell Rep. (2017) 21:1521–33. 10.1016/j.celrep.2017.10.056 29117558 PMC5695904

[17] Ley RE Turnbaugh PJ Klein S Gordon JI. Microbial ecology: human gut microbes associated with obesity. Nature. (2006) 444:1022–3. 10.1038/4441022a 17183309

[18] Ly NP Litonjua A Gold DR Celedón JC. Gut microbiota, probiotics, and vitamin D: interrelated exposures influencing allergy, asthma, and obesity? J Allergy Clin Immun. (2011) 127:1087–94. 10.1016/j.jaci.2011.02.015 21419479 PMC3085575

[19] Cantorna MT McDaniel K Bora S Chen J James J. Vitamin D, immune regulation, the microbiota, and inflammatory bowel disease. Exp Biol Med. (2014) 239:1524–30. 10.1177/1535370214523890 24668555 PMC4176535

[20] Wu GD Chen J Hoffmann C Bittinger K Chen YY Keilbaugh SA. Linking long-term dietary patterns with gut microbial enterotypes. Science. (2011) 334:105–8. 10.1126/science.1208344 21885731 PMC3368382

[21] De Vadder F Kovatcheva-Datchary P Goncalves D Vinera J Zitoun C Duchampt A. Microbiota-generated metabolites promote metabolic benefits via gut-brain neural circuits. Cell. (2014) 156:84–96. 10.1016/j.cell.2013.12.016 24412651

[22] Chang CJ Lin CS Lu CC Martel J Ko YF Ojcius DM. Ganoderma lucidum reduces obesity in mice by modulating the composition of the gut microbiota. Nat Commun. (2015) 6:7489. 10.1038/ncomms8489 26102296 PMC4557287

[23] Chang CJ Lu CC Lin CS Martel J Ko YF Ojcius DM. Antrodia cinnamomea reduces obesity and modulates the gut microbiota in high-fat diet-fed mice. Int J Obes. (2018) 42:231–43. 10.1038/ijo.2017.149 28630461 PMC5803574

[24] Solano-Aguilar GI Jang S Lakshman S Gupta R Beshah E Sikaroodi M. The effect of dietary mushroom *Agaricus bisporus* on intestinal microbiota composition and host immunological function. Nutrients. (2018) 10:1721. 10.3390/nu10111721 30424006 PMC6266512

[25] Tian Y Nichols RG Roy P Gui W Smith PB Zhang J Prebiotic effects of white button mushroom (*Agaricus bisporus*) feeding on succinate and intestinal gluconeogenesis in C57BL/6 mice. J Funct Foods. (2018) 45:223–32. 10.1016/j.jff.2018.04.008

[26] Feeney MJ Dwyer J Hasler-Lewis CM Milner JA Noakes M Rowe S. Mushrooms and health summit proceedings. J Nutr. (2014) 144:1128S−36S. 10.3945/jn.114.190728 24812070 PMC4056650

[27] Bi H Han H Li Z Ni W Chen Y Zhu J. A water-soluble polysaccharide from the fruit bodies of *Bulgaria inquinans* (fries) and its anti-malarial activity. Evid Based Complement Alternat Med. (2011) 2011:973460. 10.1093/ecam/neq070 21785644 PMC3139502

[28] Schneider K Oltmanns J Hassauer M. Allometric principles for interspecies extrapolation in toxicological risk assessment–empirical investigations. Regul Toxicol Pharmacol. (2004) 39:334–47. 10.1016/j.yrtph.2004.03.001 15135212

[29] Edgar RC. MUSCLE: multiple sequence alignment with high accuracy and high throughput. Nucleic Acids Res. (2004) 32:1792–7. 10.1093/nar/gkh340 15034147 PMC390337

[30] Borchers AT Stern JS Hackman RM Keen CL Gershwin ME. Mushrooms, tumors, and immunity. P Soc Exp Biol Med. (1999) 221:281–93. 10.3181/00379727-221-44412 10460691

[31] Jayachandran M Xiao J. Xu B. A critical review on health promoting benefits of edible mushrooms through gut microbiota. Int J Mol Sci. (2017) 18:1934. 10.3390/ijms18091934 28885559 PMC5618583

[32] Kau AL Ahern PP Griffin NW Goodman AL Gordon JI. Human nutrition, the gut microbiome and the immune system. Nature. (2011) 474:327–36. 10.1038/nature10213 21677749 PMC3298082

[33] Furusawa Y Obata Y Hase K. Commensal microbiota regulates T cell fate decision in the gut. Semin Immunopathol. (2015) 37:17–25. 10.1007/s00281-014-0455-3 25315350

[34] Oppenheim JJ. IL-2: more than a T cell growth factor. J Immunol. (2007) 179:1413–4. 10.4049/jimmunol.179.3.1413 17641004

[35] Geuking MB Cahenzli J Lawson MAE Ng DCK Slack E Hapfelmeier S. Intestinal bacterial colonization induces mutualistic regulatory T cell responses. Immunity. (2011) 34:794–806. 10.1016/j.immuni.2011.03.021 21596591

[36] Hansen JJ. Immune responses to intestinal microbes in inflammatory bowel diseases. Curr Allergy Asthma Rep. (2015) 15:61. 10.1007/s11882-015-0562-9 26306907

[37] Fung T Olson C Hsiao E. Interactions between the microbiota, immune and nervous systems in health and disease. Nat Neurosci. (2017) 20:145–155. 10.1038/nn.4476 28092661 PMC6960010

[38] Ashwini A Ramya HN Ramkumar C Reddy KR Kulkarni RV Abinaya V. Reactive mechanism and the applications of bioactive prebiotics for human health: review. J Microbiol Meth. (2019) 159:128–37. 10.1016/j.mimet.2019.02.019 30826441

[39] Wan ML Ling KH El-Nezami H Wang MF. Influence of functional food components on gut health. Crit Rev Food Sci. (2019) 59:1927–36. 10.1080/10408398.2018.1433629 29381385

[40] Laparra JM Sanz Y. Interactions of gut microbiota with functional food components and nutraceuticals. Pharmacol Res. (2010) 61:219–25. 10.1016/j.phrs.2009.11.001 19914380

[41] Chen H Mao X He J Yu B Huang Z Yu J. Dietary fibre affects intestinal mucosal barrier function and regulates intestinal bacteria in weaning piglets. Br J Nutr. (2013) 110:1837–48. 10.1017/S0007114513001293 23656640

[42] Singdevsachan SK Auroshree P Mishra J Baliyarsingh B Tayung K Thatoi H Mushroom polysaccharides as potential prebiotics with their antitumor and immunomodulating properties: a review. Bioact Carbohydr Diet Fibre. (2016) 7:1–14. 10.1016/j.bcdf.2015.11.001

[43] Xu X Zhang X. Lentinula edodes-derived polysaccharide alters the spatial structure of gut microbiota in mice. PLoS ONE. (2015) 10:e0115037. 10.1371/journal.pone.0115037 25608087 PMC4301806

[44] Mizuno M Nishitani Y Hashimoto T Kanazawa K. Different suppressive effects of fucoidan and lentinan on IL-8 mRNA expression in *in vitro* gut inflammation. Biosci Biotechnol Biochem. (2009) 73:2324–5. 10.1271/bbb.90326 19809164

[45] May RM Will a large complex system be stable? Nature. (1972) 238:413–4. 10.1038/238413a0 4559589

[46] Coyte KZ Schluter J Foster KR. The ecology of the microbiome: networks, competition, and stability. Science. (2015) 350:663–6. 10.1126/science.aad2602 26542567

[47] Zagato E Pozzi C Bertocchi A Schioppa T Saccheri F Guglietta S. Endogenous murine microbiota member *Faecalibaculum rodentium* and its human homologue protect from intestinal tumour growth. Nat Microbiol. (2020) 5:511–24. 10.1038/s41564-019-0649-5 31988379 PMC7048616

[48] Chen J Huang C Wang J Zhou H Lu Y Lou L. Dysbiosis of intestinal microbiota and decrease in paneth cell antimicrobial peptide level during acute necrotizing pancreatitis in rats. PLoS ONE. (2017) 12:e0176583. 10.1371/journal.pone.0176583 28441432 PMC5404871

[49] Cignarella F Cantoni C Ghezzi L Salter A Dorsett Y Chen L. Intermittent fasting confers protection in CNS autoimmunity by altering the gut microbiota. Cell Metab. (2018) 27:1222–35.e1226. 10.1016/j.cmet.2018.05.006 29874567 PMC6460288

[50] Liao T Chen YP Huang SQ Tan LL Li CQ Huang XA Chondroitin sulfate elicits systemic pathogenesis in mice by interfering with gut microbiota homeostasis. bioRxiv. (2017). 10.1101/142588

[51] Wells JM Rossi O Meijerink M van Baarlen P. Epithelial crosstalk at the microbiota–mucosal interface. Proc Natl Acad Sci USA. (2011) 108 (Suppl. 1):4607–14. 10.1073/pnas.1000092107 20826446 PMC3063605

[52] Hooper LV Littman DR Macpherson AJ. Interactions between the microbiota and the immune system. Science. (2012) 336:1268–73. 10.1126/science.1223490 22674334 PMC4420145

[53] Manichanh C Borruel N Casellas F Guarner F. The gut microbiota in IBD. Nat Rev Gastroenterol Hepatol. (2012) 9:599–608. 10.1038/nrgastro.2012.152 22907164

