# Supplementary figures and images for: Mushroom Bulgaria inquinans Modulates Host Immunological Response and Gut Microbiota in Mice
Source: Front Nutr. 2020 Oct 8;7:144. doi: 10.3389/fnut.2020.00144 (PMC7578393; doi:10.3389/fnut.2020.00144)

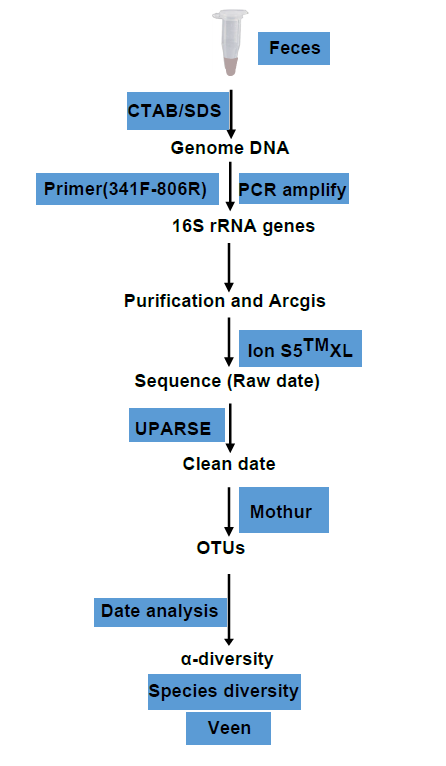
**Supplementary Figures**

**F****igure 1.** Flow diagram of 16sRNA.

Supplement: Supplementary file 1 [file Table_1.DOCX]
